# ID 70 - Uso de Alfaporactanto no Tratamento de Doenças da Membrana Hialina em Neonatos Comparado ao Beractanto: revisão sistemática da literatura e impacto orçamentário

**DOI:** 10.5327/2237-9622.2025.v34s1.70

**Published:** 2025-11-25

**Authors:** Ellen Daiane Biavatti de Oliveira Algeri, Iara Beatriz Andrade Sousa, Dayse Sanches Guimarães Paião, Rita de Cássia Dorácio Mendes, Israel Moraes Santos, Gilson Dorneles, Alessandro Teixeira Andrade

**Keywords:** tecnologia em saúde, surfactante, doença da membrana hialina, intensivismo neonatal

## Abstract

**Introdução:**

A doença da membrana hialina (DMH) é uma complicação que acomete recém-nascidos prematuros afetando os pulmões pela falta de surfactante. Recomenda-se, como tratamento, a terapia de reposição de surfactante de origem exógena, por reduzir a doença em 50% e a mortalidade neonatal em 30%. Especialista da Unidade de Terapia Intensiva Neonatal (Utin) do HU-UFGD/Ebserh solicitaram a padronização do Alporactanto justificando maior eficácia e segurança e menor número de doses adicionais em neonatos prematuros quando comparado ao Beractanto. O objetivo foi analisar a eficácia e a segurança do uso de Alfaporactanto no tratamento da DMH em neonatos comparado ao Beractanto.

**Método:**

Revisão Sistemática de Ensaios Clínicos Randomizados com buscas nas bases: MEDLINE, Embase, LILACS, base de registro: Clinical Trials e buscas complementares em websites de agências de Avaliação de Tecnologias em Saúde e instituições correlatas. Foram considerados os desfechos de mortalidade e displasia broncopulmonar para eficácia e segurança dos tratamentos, respectivamente. A avaliação do risco de viés foi realizada por meio da ferramenta RoB 2.0, e a certeza no conjunto final da evidência foi avaliada utilizando a abordagem GRADE. Realizou-se a análise do Impacto Orçamentário Incremental baseado no consumo da tecnologia padronizada na instituição (Beractanto) no período de um ano, considerando a demanda aferida na Utin da instituição.

**Resultados:**

Foram incluídas na revisão oito estudos com 965 participantes, com variação de tamanho amostral de 52 a 197. Para o desfecho óbito, houve redução de 39% do risco na intervenção, com imprecisão dos resultados devido à inconsistência entre os estudos. O estudo de Lemyre (2017) apresentou 21,4% de mortalidade do Alfaporactanto e 6,7 % do Beractanto (9,4% foram por infecção hospitalar não associada a complicações da prematuridade). Quanto a dosagens adicionais, no estudo de Ramanathan (2004), 73% foram tratadas com sucesso com apenas uma dose de 200 mg/kg de Alfaporactanto em comparação com 51% no grupo Beractanto 100 mg/kg, indicando possível heterogeneidade clínica entre os estudos. Dizdar (2012) demonstra que o grupo que recebeu Beractanto necessitaram de duas doses em comparação com o grupo que recebeu o Alfaporactanto, (31% vs 12%, respectivamente) o que impacta tanto do custo-efetividade devido ao valor unitário da tecnologia e no volume administrado no pulmão do neonato via tubo orotraqueal. Para o desfecho de displasia broncopulmonar, os estudam apontam redução significativa (31%) de eventos relacionados ao uso do Alfaberactanto sugerindo potencial segurança do tratamento em relação ao comparador. O risco de viés geral dos estudos indicou algumas preocupações. A certeza no conjunto final da evidência foi considerada baixa, uma vez que o intervalo de confiança de todos os estudos é amplo gerando inconsistência e imprecisão. O Impacto Orçamentário Incremental apontou acréscimo de R$ 56.379,84 anual, ou seja, um acréscimo de R$ 247,28 ao ano, por neonato.

**Conclusão:**

Os nossos achados indicam similaridade de eficácia entre as intervenções, apesar da baixa certeza da evidência que sugere que estudos futuros poderão modificar a direção e a magnitude de efeito. Em relação à segurança houve menor número de eventos de displasia pulmonar e menor necessidade de doses adicionais no uso do Alfaporactanto quando comparado ao de Beractanto. O Nats emitiu a recomendação condicional a favor da tecnologia.

